# PPAR*γ* Agonists: Blood Pressure and Edema

**DOI:** 10.1155/2010/785369

**Published:** 2009-12-22

**Authors:** Bonnie L. Blazer-Yost

**Affiliations:** Department of Biology, Indiana University-Purdue University Indianapolis, 723 West Michigan Street, SL 358 Indianapolis, IN 46202, USA

## Abstract

Peroxisome proliferator activated receptor *γ* (PPAR*γ*) agonists are widely used in the treatment of type 2 diabetes. Side effects of drug treatment include both fluid retention and a lowering of blood pressure. Data from animal and human studies suggest that these effects arise, at least in part, from drug-induced changes in the kidney. In order to capitalize on the positive aspect (lowering of blood pressure) and exclude the negative one (fluid retention), it is necessary to understand the mechanisms of action underlying each of the effects. When interpreted with known physiological principles, current hypotheses regarding potential mechanisms produce enigmas that are difficult to resolve. This paper is a summary of the current understanding of PPAR*γ* agonist effects on both blood pressure and fluid retention from a renal perspective and concludes with the newest studies that suggest alternative pathways within the kidney that could contribute to the observed drug-induced effects.

## 1. Introduction

PPAR*γ* agonists, also called thiazolidinediones (TZDs), are widely used as insulin-sensitizing agents in the treatment of type 2 diabetes. One unanticipated, and poorly understood, side effect of the TZDs is a lowering of blood pressure. In the vast majority of patients treated with these drugs, the change in blood pressure per se can be viewed as a positive consequence of drug therapy. Statistically the classic type 2 diabetic patient has increased blood pressure that is treated by pharmaceutical intervention. Indeed, this beneficial property as well as positive effects on lipid profiles and inflammatory responses has prompted the suggestion that this class of drugs might be useful in treating metabolic syndrome, a prediabetic state that is reaching epidemic proportions in industrialized societies [1, 2]. 

 However, treatment with PPAR*γ* agonists is not without negative side effects. Particularly noteworthy when considering using TZDs to treat overweight or obese patients is the propensity for these drugs to cause weight gain. The weight gain is multifactorial and includes a positive effect on adipogenesis [3] and a diuretic-resistant fluid retention [4, 5]. As with the effect on blood pressure, the physiological mechanisms responsible for fluid retention are poorly understood. 

 This paper examines the positive (lowering of blood pressure) and the negative (fluid retention) side effects of TZD treatment from a renal perspective. An important consideration for the development of drugs to treat metabolic syndrome is the question of whether the beneficial side effect can be dissociated from the negative action. Unfortunately, at this point one can only outline the issues—the answers await further experimentation to elucidate the mechanisms of action involved in these side effects.

## 2. Fluid Retention

The PPAR*γ* agonist-induced fluid retention results in plasma volume expansion (often measured as a decrease in hematocrit) and peripheral edema [5]. The excess fluid retention is relatively resistant to diuretics [4, 5]. In a diuretic comparison study, the most promising results were obtained with intensive therapy using an aldosterone antagonist that has actions in the distal tubule/collecting duct [6]. 

 The propensity to cause fluid retention is serious enough to raise questions about the continued use of these agonists—particularly in a marginal patient population. Rat studies have indicated that the increased plasma volume can cause relatively rapid cardiac remodeling, even in healthy animals [7]. A recent high-profile meta-analysis has indicated that Avandia (rosiglitazone) increases the risk of death from cardiovascular disease [8], while other studies have observed beneficial effects of TZDs on major cardiovascular events in humans as well as protection against ischemia-reperfusion injury and reduction of myocardial infarct size in animal models [9–11]. Additional data are required to ascertain the risk/benefit relationships of TZD therapy for patients with cardiovascular risk factors but it is clear that edema is an undesirable side effect. Effective therapy to limit fluid retention would increase the usefulness of this class of compounds and is necessary if they are to be used as a prophylactic treatment to delay the progression of metabolic syndrome to type 2 diabetes.

## 3. Blood Pressure

Modest decreases in blood pressure during treatment with PPAR*γ* agonists are a consistent finding in studies conducted in normal, diabetic, and hypertension-prone rodents and humans (see [1]). The effect, when measured continuously in rodents, is rapid and usually manifested within 12–24 hours of the initial dosing [7, 12]. Is this a secondary side effect of the drugs per se or does this observation indicate that the receptor is important in maintaining a normal blood pressure? An experiment of nature indicates the latter. Barroso et al. characterized rare dominant negative mutations in the human peroxisome proliferator activated receptor *γ*. As expected, these patients have insulin resistance and diabetes mellitus. Interestingly, the patients also have severe hypertension that is difficult to control [13]. Thus, the data indicate that loss of the receptor leads to severe increases in blood pressure and activation of the receptor as seen in PPAR*γ* agonist therapy causes a decrease in blood pressure.

## 4. Site of Action

There is general consensus that the change in blood pressure is likely due to a combination of renal and vascular effects. This contention is underlined by the observations that TZDs simultaneously increase fluid retention and decrease blood pressure. Assuming normal cardiac function, it is hard to imagine a scenerio where plasma volume expansion leads to a decrease in blood pressure without a substantial change in the vasculature. 

 PPAR*γ* regulation in the vasculature is complex and may exert actions on both endothelial and smooth muscle cell function. Effects on the multiple parameters of vascular function have been recently elucidated using vascular cell-type-specific PPAR*γ* knockout mice. Animals defective in the endothelial receptor are hypertensive, an effect that the authors linked to PPAR*γ* regulation of nitric oxide production in this cell type [14]. Conversely, a vascular smooth muscle-selective PPAR*γ* knockout mouse resulted in an animal displaying a hypotensive phenotype [15]. The authors have shown that the mechanism for the change in blood pressure regulation is due to a PPAR*γ* regulation of *β*2-adrenergic receptor expression and concomitantly a change in *β*-adrenergic agonist sensitivity. Wang and co-authors used knockout mice to examine both endothelial and vascular smooth muscle effects of PPAR*γ* and concluded that there were distinct functions for the PPAR*γ* in both cell types but that the endothelial regulation was responsible for the blood pressure lowering effects of PPAR*γ* agonists [16]. In addition, high levels of TZDs appear to increase vascular permeability via a variety of proposed mechanisms including vascular endothelial growth factor, nitric oxide, and protein kinase C (reviewed in [17]). These recent studies highlight the complexity of the vascular regulation. The remainder of this paper will focus on the initial TZD-mediated changes that are manifested in the kidney, specifically the collecting duct. 

 Two separate knockout technologies were exploited to specifically ablate the PPAR*γ* in the renal collecting duct of mice [18, 19]. In the absence of the collecting duct receptor, the mice did not show the typical fluid retention when treated with clinically used TZDs. In contrast, the normal littermates showed reduced hematocrits, and fluid derived weight gain after treatment. These studies substantiate the notion that the collecting duct plays a primary role in the PPAR*γ*-mediated fluid retention. Unfortunately, continuous monitoring of blood pressure was not reported in these studies.

## 5. Mechanism of Action: The ENaC Hypothesis

The collecting duct knockout animal studies were the “icing on the cake” for an emerging hypothesis to explain how PPAR*γ* agonists cause fluid retention. The principal cells lining the distal tubule and collecting duct are the site of hormonally regulated Na^+^ transport. These hormones regulate whole body salt and water homeostasis and, therefore, blood pressure. Principal cells respond to steroid (aldosterone) and peptide (antidiuretic hormone, ADH; insulin/IGF1) hormones with an increase in Na^+^ reabsorption leading to increased plasma volume. All three hormones exert their effects via an insertion of the epithelial Na^+^ channel (ENaC) into the luminal plasma membrane of the principal cells thereby initiating the Na^+^ resorptive cascade [20–24]. Thus, it is logical to hypothesize that any agent that causes salt and water retention might have an effect in the distal nephron, specifically on ENaC. In addition, the PPAR*γ* is expressed in the distal tubule and collecting duct. 

 Studies prior to the creation of the PPAR*γ* collecting duct-specific knockout mice demonstrated a remarkable degree of Na^+^ and fluid retention and biochemical changes consistent with ENaC activation. Song et al. found that normal Sprague-Dawley rats fed a high dose of rosiglitazone (94 mg/kg body weight) exhibited a 22% decrease in urine volume and a 44% decrease in Na^+^ excretion [12]. Chen et al. found that GI262570, a PPAR*γ* agonist, changed electrolyte and water reabsorption in the distal nephron of Sprague-Dawley rats [25]. Hong et al. [26] demonstrated that PPAR*γ* activation increased the cell surface expression of the *α* subunit of ENaC by upregulating serum glucocorticoid kinase (SGK), an enzyme previously shown to be a convergence point for hormone activation of ENaC [27, 28]. All of these findings are consistent with a TZD effect on ENaC or pathways that regulate this Na^+^ channel. Further corroboration was found in studies demonstrating that amiloride (a specific inhibitor of ENaC) prevented the TZD-induced increase in body weight gain in mice [19] and the previously cited finding that an aldosterone antagonist, which acts to supress ENaC activity, is the most effective agent for combating PPAR*γ* agonist-induced fluid retention [6]. From all of these early studies, it appeared that upregulation of ENaC was responsible for the PPAR*γ* agonist-induced salt and water retention. 

 In the myriad of studies that followed the initial findings, some discrepancies began to appear. For example, not all studies were able to demonstrate inhibition of fluid retention by amiloride. There was a lack of consistency between studies as to which, if any, of the three ENaC subunits was regulated by the agonists and whether SGK actually changed in response to treatment. Nofziger et al. were unable to reproduce the stimulation of ENaC by PPAR*γ* agonists in any of three well-characterized principal cell lines that endogenously express the receptor [29]. In human and animal studies, there did not appear to be a consensus as to the effect of TZDs on aldosterone secretion. In some studies this hormone level has been reported to increase [7, 30], and in others PPAR*γ* agonist treatment resulted in a decreased aldosterone level [6, 12, 18, 25].

## 6. Mechanism of Action: Physiological Considerations

While the ENaC hypothesis is appealing in its simplicity, is this the whole story? Do all of the findings correlate with known physiological principles? First and foremost, does the correlation between increased fluid retention and decreased blood pressure make sense, particularly when evoking regulation via an aldosterone/ENaC mechanism? 

 Amiloride and aldosterone antagonists are clinically used diuretics that inhibit ENaC activity directly or secondarily, and lower blood pressure by *decreasing* Na^+^ and fluid retention. In the treatment of hypertension, an increase in Na^+^ reabsorption via ENaC is equated with an increase in blood pressure not a decrease as seen during PPAR*γ* agonist therapy. 

 There are some interesting experiments of nature that can inform this issue as well. There are human mutations in ENaC subunits that give rise to both gain-of-function and loss-of-function in channel plasma membrane expression or activity. The gain-of-function mutations known as Liddle's syndrome result in severe, diuretic-resistant hypertension [31]. In loss-of-function mutations, life-threatening salt wasting, hypotension, and hyperkalemia occur during the neonatal period [32, 33]. Based on the presentations of these naturally occuring mutations, which alter ENaC activity in an in vivo setting, one can conclude that it is unlikely that PPAR*γ* agonist stimulation of ENaC will result in a decreased blood pressure. Thus, physiological principles as we understand them seem inadequate to explain how ENaC stimulation and increased fluid retention correspond to a decrease in blood pressure. 

 In a recent study, Vallon and colleagues [34] conditionally inactivated the *α* subunit of ENaC in the collecting duct of mice. This technique functionally inactivates the Na^+ ^ channel in the same area of the kidney as the previous PPAR*γ* depletion experiments. The mice in which collecting duct ENaC was inactivated still demonstrated the PPAR*γ* agonist fluid retention as observed in the control mice. Blood pressure measurements were not reported in this study. The authors concluded that activation of ENaC is not the primary mechanism in the TZD-induced fluid retention. 

## 7. Mechanism of Action: Alternative Possibilities

If the ENaC hypothesis does not fit well with known physiological principles, are there alternative possibilities? Putting all the data together, it does appear that one of the sites of action for both fluid retention and blood pressure effects is indeed the kidney, specifically the collecting duct. The strongest support for this contention results from the collecting duct-specific PPAR*γ* knockout mice [18, 19]. If the primary effect is not manifested on ENaC, it is likely that the observed changes in Na^+^ retention are secondary consequences in response to changes in other ion fluxes. Such secondary effects would explain the diuretic efficacy and also the variability seen in hormonal responses and changes in ENaC subunit abundance. 

 When examining other potential ion transporters, one intriguing possibility is a TZD-mediated change in Cl^−^. Diets contain approximately equal concentrations of Na^+^ and Cl^−^ but the movement of Na^+^ is considered to be of primary importance because it is hormonally controlled. However, there are emerging studies that suggest that control of renal Cl^−^ transport can modulate blood pressure as well. 

 A number of different chloride channels have been found in various segments of the renal nephron [35, 36]. Mutations in one of these channels, termed ClCK-Kb, has been shown to predispose carriers to hypertension [37], leading to the conclusion that this channel may be relevant in salt sensitivity of blood pressure regulation [38]. One of the major Cl^−^ channels found in the collecting duct is the cystic fibrosis transmembrane regulator (CFTR) [35]. CFTR is important for maintaining fluid balance, particularly in the lung, as demonstrated by the loss of this channel in cystic fibrosis. While changes in the activity of Cl^−^ channels in other portions of the nephron have been linked to changes in blood pressure, a role for the CFTR Cl^−^ channel has yet to be established. Tantalizingly, like ENaC, this channel is activated by ADH and could, theoretically, be involved in hormonal control of salt and water balance in this portion of the nephron [35, 39, 40]. 

 We have recently found that a variety of PPAR*γ* agonists inhibit ADH-stimulated Cl^−^ secretion in a cell culture model of the principal cells of the cortical collecting duct. The potency of the agonists to inhibit hormone-stimulated Cl^−^ transport mirrors receptor transactivation profiles except that the IC_50_ for the effect on Cl^−^ transport is an order of magnitude more sensitive. Analyses of the components of the ADH-stimulated intracellular signaling pathway indicate no PPAR*γ* agonist-induced changes in any of the known steps in the transepithelial signaling pathway. The PPAR*γ * agonist-induced decrease in anion secretion is the result of a decrease in the mRNA encoding the final effector in the pathway, the apically located CFTR Cl^−^ channel [40]. These data showing that CFTR is a target for PPAR*γ* agonists provide new theoretical possibilities for PPAR*γ* agonist-induced fluid retention. The data, if substantiated by in vivo experiments, indicate that CFTR plays a role in fluid balance. 

 Recently, a similar PPAR*γ* agonist-mediated effect on Cl^−^ flux has been demonstrated in mouse intestinal epithelia. Mice fed with rosiglitazone for 8 days had reduced intestinal forskolin-stimulated anion secretion and substantially inhibited cholera toxin-induced intestinal fluid accumulation. Both of these processes occur via CFTR mechanisms. In the HT29 intestinal cell line, 5-day treatment with rosiglitazone inhibits cAMP-dependent Cl^−^ secretion. This decreased secretion was accompanied by decreases in the protein levels of CFTR, the Na^+^/K^+^/2Cl^−^ cotransporter that allows basolateral influx of Cl^−^ and in KCNQ1, which serves as a basolateral K^+^ recycling channel [41]. 

 Thus, there are data to suggest that the primary effect of PPAR*γ* agonists on ion transporters in polarized epithelial cells is due to a decrease in the expression of the CFTR channel and, perhaps, in other transporters that mediate Cl^−^ secretion. Exactly how this translates into simultaneous effects on fluid retention and decreased blood pressure is unknown. The theoretical possibilities range from changing electrochemical driving forces for transepithelial transport to altering ion and fluid partitioning between the interstitium and the vascular fluid compartment.

## 8. Summary

While the mechanisms of action of the PPAR*γ* agonists in lowering blood pressure and causing fluid retention remain unknown, several important concepts are beginning to emerge. The most recent data suggest that the TZDs do not directly regulate the ENaC although changes in ENaC-mediated Na^+^ transport may occur secondarily. Until the physiological mechanisms of action leading to the TZD side effects are fully understood, drugs such as mineralocorticoid antagonists and ENaC channel blockers may be the most effective diuretics in the treatment of PPAR*γ*-mediated edema. However, for development of new drug therapies devoid of the negative side effect of fluid retention, the biochemical mechanism of action of the TZDs on renal and vascular ion and water channels must be more thoroughly investigated.
